# Micro-/Nanofiber Optics: Merging Photonics and Material Science on Nanoscale for Advanced Sensing Technology

**DOI:** 10.1016/j.isci.2019.100810

**Published:** 2019-12-28

**Authors:** Lei Zhang, Yao Tang, Limin Tong

**Affiliations:** 1 State Key Laboratory of Modern Optical Instrumentation, College of Optical Science and Engineering, Zhejiang University, Hangzhou 310027, China

**Keywords:** Fiber Optics, Nanomaterials, Nanostructure

## Abstract

Micro-/nanofibers (MNFs) are optical fibers with diameters close to or below the wavelength of the guided light. These tiny fibers can offer engineerable waveguiding properties including optical confinement, fractional evanescent fields, and surface intensity, which is very attractive to optical sensing on the micro-/nano scale. In this review, we first introduce the basics of MNF optics and MNF optical sensors from physical and chemical to biological applications and review the progress and current status of this field. Then, we review and discuss hybrid MNF structures for advanced optical sensing by merging MNFs with functional structures including chemical indicators, quantum dots, dye molecules, plasmonic nanoparticles, 2-D materials, and optofluidic chips. Thirdly, we introduce the emerging trends in developing MNF-based advanced sensing technology for ultrasensitive, active, and wearable sensors and discuss the future prospects and challenges in this exciting research field. Finally, we end the review with a brief conclusion.

## Graphical Abstract

**Figure:**
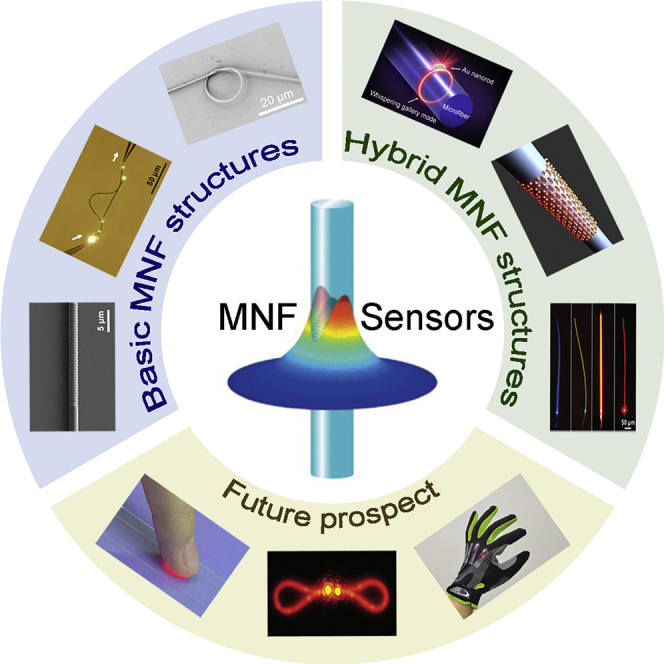

## Introduction

In the past decades, optical fiber sensor has been one of the most successful and powerful applications of both fiber optics and sensing technology (Lee, 2003, Udd and Spillman, 2011). Recently, along with the rapid progress in nanotechnology, biology, and increasing demands on optical sensors with higher performances and versatilities, size miniaturization has been one of the current trends of fiber-optic sensors (Ferreira et al., 2017). It is obvious that a compact sensing structure can bestow the sensor with faster response, higher sensitivity, low power consumption, and better spatial resolution, and an optical micro-/nanofiber (MNF) with diameters close to or below the vacuum wavelength of visible or near-infrared light is one of the best candidates for this purpose (Brambilla et al., 2009, Tong and Sumetsky, 2011).

As a combination of fiber optics and nanotechnology, the optical MNF (also called optical fiber nanowires and microwires, nanofiber or nanotaper when its diameter is below 1 μm) has been emerging as a novel platform for exploring fiber-optic technology on the micro- or nanoscale (Guo et al., 2014, Ismaeel et al., 2013, Tong et al., 2012, Wu and Tong, 2013). Fabricated by taper-drawing technique, an MNF shows uniform diameter, smooth sidewall, and outstanding mechanical flexibilities (Tong et al., 2003). With high-index contrast between the MNF material (e.g., glass or polymer) and the surroundings (e.g., air or water), MNF guides light with low optical loss, tight optical confinement, and large fractional evanescent fields (Tong et al., 2004), making it a novel miniaturized platform for optical sensing with special advantages including faster response, higher sensitivity, higher spatial resolution, and lower-power operation.

To data, a variety of physical, chemical, or biological optical MNF sensors have been demonstrated (Chen et al., 2018a, Chen et al., 2019, Guan and Huang, 2019, Kou et al., 2012, Lou et al., 2014, Talataisong et al., 2018, Tong, 2018, Wang et al., 2015a, Wu et al., 2018, Yan and Xu, 2017). Here we review the recent progress in optical MNF sensors regarding their fabrication, waveguide properties, and sensing applications. Basic MNF structures (e.g., biconical MNFs, MNF couplers, Mach-Zehnder interferometers, optical gratings, and circular microcavities) and hybrid MNF structures (e.g., functionalized polymer MNFs, metallic-nanostructure-activated MNFs, graphene-decorated MNFs, and optofluidic MNFs) for advanced optical sensing are summarized. At the same time, we will discuss prospects and challenges of MNF optical sensors to some extent, with several clues for further studies, including ultrasensitive optical force sensors at nanoscale, ultrasensitive biosensing based on plasmonics or optofluidic biolasers and wearable optical sensors for human health monitoring. Finally, we end the article with a brief conclusion.

### Fabrication and Manipulation of MNFs

For optical waveguiding, excellent geometric uniformity and surface smoothness of the MNFs is critical for achieving low optical loss and high signal-to-noise ratio, as well as tightly confined large fractional evanescent fields, and therefore the fabrication process of these tiny fibers is vitally important. To bestow the as-fabricated MNFs with greater versatilities, a number of post-fabrication techniques have been reported in the past years. In this section, we briefly review taper-drawing techniques for fabricating and manipulating MNFs.

### Fabrication of Silica MNFs

**Figure 1:**
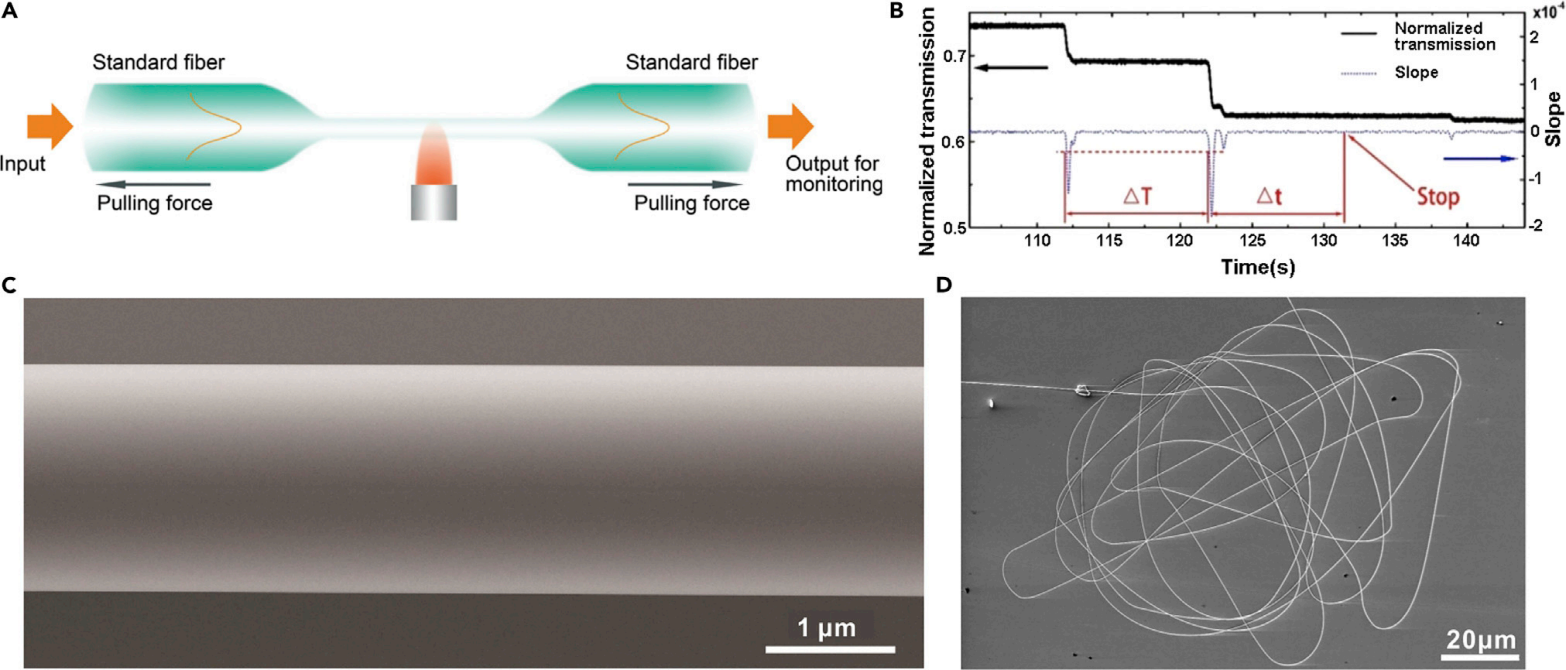
Fabrication of Silica MNFs (A) Schematic diagram of flame-heated taper drawing of an MNF from a standard optical fiber. Light is launched into and guided through the fiber and the MNF for in-situ monitoring by measuring the transmission behavior of the MNF. Reproduced with permission from Wu and Tong (Wu and Tong, 2013). Copyright 2013 Science Wise Publishing & DE GRUYTER. (B) The normalized transmission curve of 785 nm laser as a function of time during the tapering process. Reprinted with permission from Xu et al. (Xu et al., 2017). Copyright 2017 Optical Society of America. (C) SEM image of a typical as-drawn silica microfiber, showing excellent diameter uniformity and sidewall smoothness. Reproduced with permission from Wang et al. (Wang et al., 2015b). Copyright 2015 American Chemical Society. (D) SEM image of a 4-mm-long wire with a diameter of 260 nm. Reproduced with permission from Tong et al. (Tong et al., 2003). Copyright 2003 Nature Publishing Group.

Flame-heated taper drawing is mostly used to draw silica MNFs from standard optical fibers. A typical illustration of flame-heated taper-drawing process is shown in Figure 1A. A hydrogen flame is used for heating the fiber. Under a certain pulling force, the fiber is stretched and elongated gradually with reduced diameter until the desired length or diameter of the fiber taper is reached. Because the as-fabricated MNF is usually attached to the standard fiber through the tapering area at both ends, it is usually mentioned as a “biconical” fiber taper or MNF. By real-time measuring optical transmission via standard fiber at both ends, it is possible to *in-situ* monitor the waveguiding properties of the MNF during the pulling process in terms of propagation loss, multi-mode interference, and group velocity delay (Boucouvalas and Georgiou, 1985, Orucevic et al., 2007). Based on the taper-drawing process mentioned above, in recent years, a number of improvements on this technique have been reported for fabricating MNFs with various parameters including ultrasmall diameters (Tong et al., 2005b), optimized tapering profiles (Xuan et al., 2010b), controllable cross-section geometries (Pricking and Giessen, 2010, Xuan et al., 2010b), and reduced propagation losses (Hoffman et al., 2014). For example, recently, with an elaborately designed taper-drawing system with feedback from the *in-situ* transmission drop due to the cut-off of the high-order waveguiding modes, Xu et al. demonstrated the possibility of drawing an MNF with precisely controlled diameter (deviation <5 nm) (Xu et al., 2017).

In some situations, conventional flame-heated systems may present disadvantages such as the random turbulence of the flame and oxygen requirement in the burning process, leading to H_2_O/OH contamination in MNFs. To avoid these issues, electrically heated taper-drawing approach is a simple and effective technique for fabricating high-quality silica MNFs (Brambilla et al., 2005, Coillet et al., 2010, Shi et al., 2006). Usually, the electrical heater can be shaped into various geometries to precisely generate required temperature and temperature distribution, which makes it possible to draw MNFs with more flexibilities. Moreover, by exempting the flame and air flow, this technique can be conducted in desired atmosphere including vacuum and thus avoid H_2_O/OH or other contamination from surrounding environment. Besides the above-mentioned techniques, a CO_2_ laser beam can also be used as an alternative heating source. By drawing MNFs in a microfurnace comprising a sapphire tube heated with a CO_2_ laser, Sumetsky successfully fabricated sub-μm-diameter MNF with excellent surface smoothness and diameter uniformity (Sumetsky et al., 2004).

### Fabrication of Polymer MNFs

**Figure 2:**
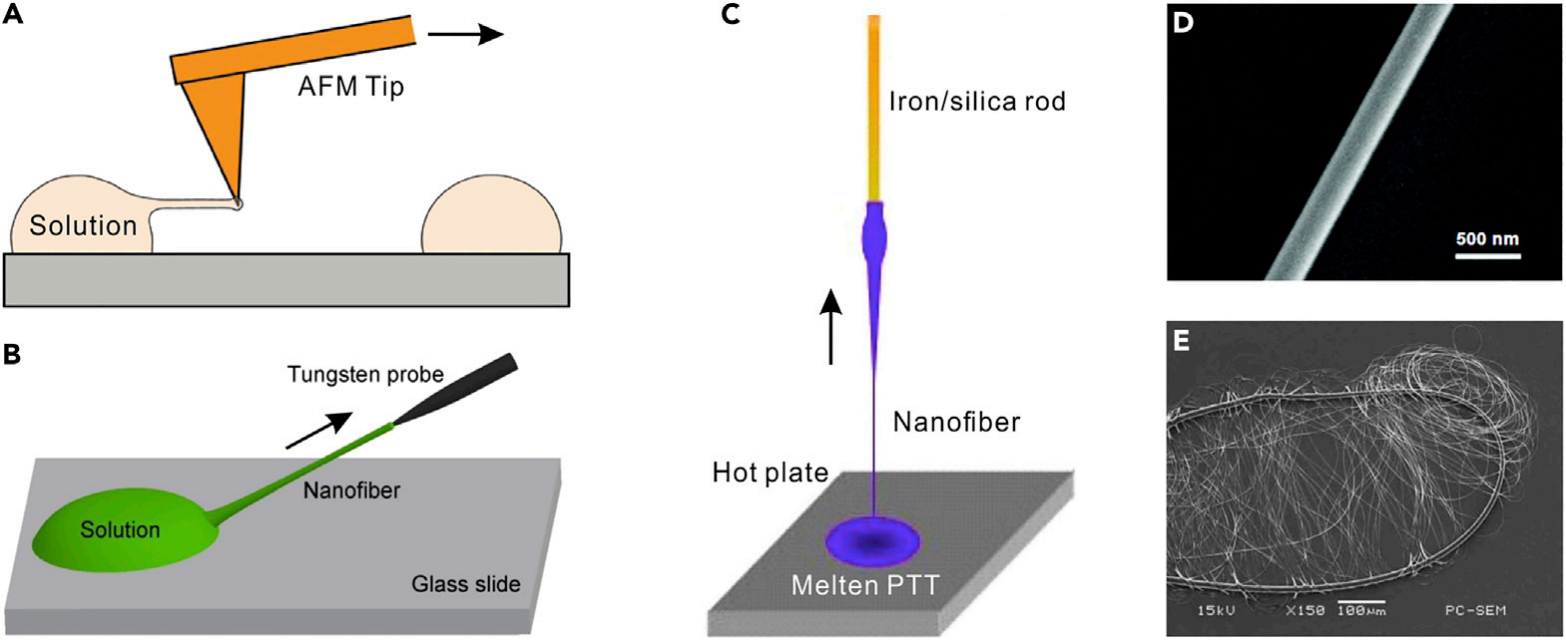
Fabrication of Polymer MNFs (A and B) Schematic illustration of drawing nanofiber from polymer solution by using AFM tip (A) and tungsten probe (B). Reproduced with permission from Harfenist et al. (Harfenist et al., 2004). Copyright 2004 American Chemical Society. (C) Schematic illustration of drawing nanofiber from molten PTT. (D) SEM image of a 290-nm-diameter BTB-doped PMMA nanofiber. (E) SEM image of coiled nanofiber with a length of about 200 mm and an average diameter of 280 nm. Reprinted with permission from Xing et al. (Xing et al., 2008). Copyright 2008 Optical Society of America.

For polymer MNFs, a number of techniques, including chemical synthesis (Cui et al., 2006), nanolithography (De Marco et al., 2008), electrospinning (Dzenis, 2004), and physical drawing (Gu et al., 2008, Harfenist et al., 2004, Xing et al., 2008) have currently been developed for the fabrication of polymer nanofibers. Among these techniques, physical drawing is an optimal method for fabricating polymer nanofibers with excellent surface qualities that are highly desired for low-loss optical waveguiding. In a typical physical drawing fabrication, a sharp tip (e.g., an AFM tip (Figure 2A) or a tungsten probe (Figure 2B) or an iron/silica rod (Figure 2C)) is used to directly draw polymer nanofibers out of a droplet of polymer solution or melt polymer onto a glass slide. Using this technique, optical-quality polymer nanofibers of polystyrene (PS), poly(methyl methacrylate) (PMMA), polyacrylamide (PAM), poly(vinyl alcohol) (PVA), poly(ethylene oxide) (PEO), and poly(trimethylene terephthalate) (PTT) have been fabricated with high uniformity and excellent surface smoothness (Figures 2D and 2E). Usually, the diameter of the as-drawn polymer nanofibers can be roughly controlled by the drawing speed and the solution concentration. It is worth noting that compared with physical drawing approach, electrospinning is much convenient for high-volume production of polymer MNFs from a broad range of polymer materials. Additionally, the electrospun polymer MNFs can be directly collected as uniaxially aligned arrays by properly designing the conductive collector (Li et al., 2004), making it possible to pattern the nanofibers during the fabrication process.

### Manipulation of As-Fabricated MNFs

To integrate as-fabricated silica or polymer MNFs into nanophotonic circuits or devices, micromanipulation techniques are desired for tailoring and assembling these tiny building blocks into functional structures or geometries (Brambilla et al., 2009, Ismaeel et al., 2013, Tong et al., 2005a). Using precisely controlled tungsten or tapered fiber probes with tip sizes of tens to hundreds of nanometers, polymer nanofibers can be cut, picked up, transferred, bent, and shaped under an optical microscope. To intercept a section of polymer nanofiber with a desired length from a polymer nanofiber, an electrochemically sharpened tungsten probe mounted on a three-dimensional translation stage is used to cut the MNF at the desired point. The intercepted MNF is then picked up, transferred, and deposited onto a certain substrate (e.g., a low-index MgF_2_ wafer or silica aerogel) using a tapered fiber probe for nanophotonic integration. When deposited on a certain substrate with a smooth surface, MNF can be firmly held in position by van der Waals forces and electrostatic interactions between the nanofiber and the substrate. Using directional pushing or dragging operations on the substrate surface against the friction force with micromanipulation probes, polymer nanofibers can be bent and assembled into desired structures or patterns. Besides, to bestow the as-fabricated MNFs with greater versatilities, a number of post-fabrication techniques including plastic bend (Tong et al., 2005a), coating (Villatoro and Monzón-Hernández, 2005, Zhang et al., 2008), embedding (Lou et al., 2010, Vienne et al., 2007, Xiao et al., 2009, Xiao et al., 2011a, Xu and Brambilla, 2007), and fusion splicing (Jin et al., 2016, Li et al., 2011, Liu et al., 2015, Pal and Knox, 2009, Wang et al., 2010) of MNFs have been investigated. So far, a variety of MNF-based functional structures including microcouplers, resonators, interferometers, and loop mirrors have been experimentally realized, which have added new possibilities for MNF sensors, as discussed later.

### Packaging of As-Fabricated MNFs

To develop robust and functional sensors based on MNFs, adequate and effective protection is highly desired because the surface contamination and environmental factors are likely to affect the response and stability of the MNF sensors when exposed to ambient air. Packaging the MNF sensors is an effective way to improve the sensor's stability, which is critical for practical applications. Note that the encapsulation of the MNFs must not sacrifice their large fractional evanescent fields, low-loss optical transmission, and excellent mechanical properties. So far, several groups have reported different embedded MNF sensors by using low RI polymers, such as PDMS (Polynkin et al., 2005), Teflon (Xu and Brambilla, 2007, Lou et al., 2010), EFIRON PC-373 (Vienne et al., 2007), and Nafion (Cai et al., 2019). In addition, silica aerogel (Xiao et al., 2009) is another promising material for embedding MNFs owing to its very low RI (n = 1.05), gas-permeability, and hydrophobicity. Compared with the MNF sensors using free-standing or substrate-supported MNFs, the packaged MNF sensors show enhanced portability, robustness, and long-term stability. For example, Zhang et al. reported a highly sensitive temperature sensor based on a coupled SU-8 resonator-MNF system. Because the MNF and the resonator are packaged by PDMS, the sensor shows no evident degradation after one-year operation (Zhang et al., 2019b).

### Basic MNF Optics for Optical Sensing

#### Waveguiding Modes in MNFs

**Figure 3:**
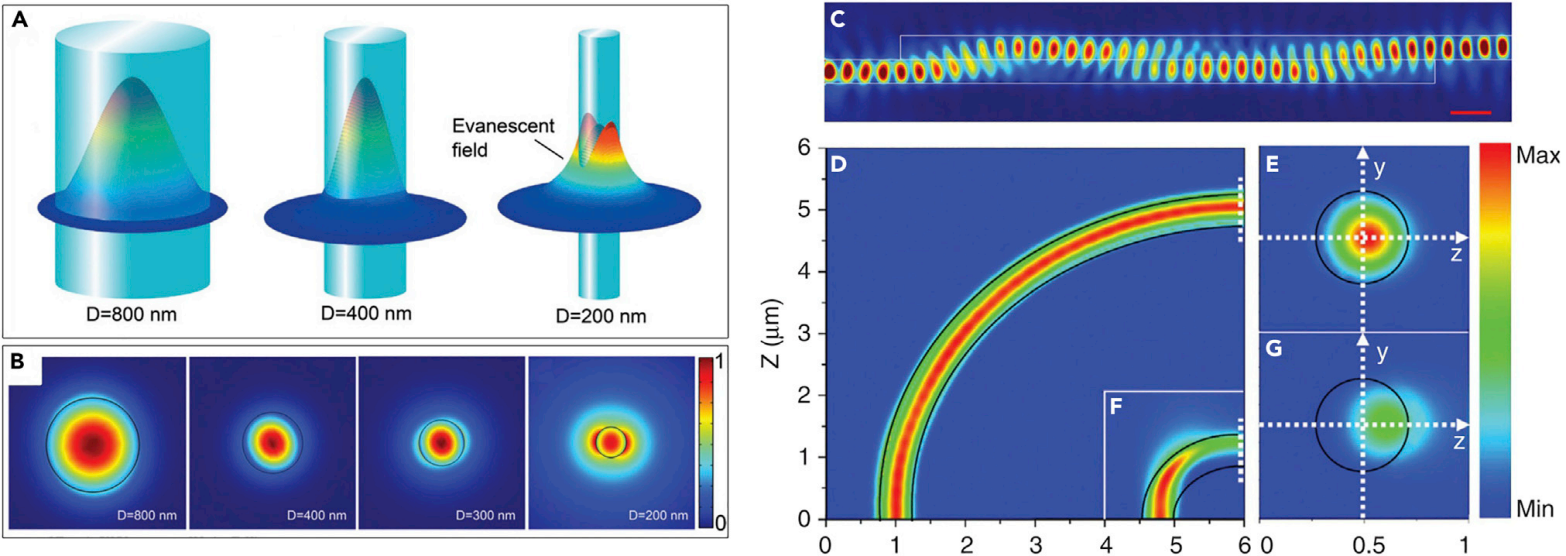
Optical Properties of Waveguiding MNFs (A and B) Z-direction Poynting vectors of silica MNFs at 633-nm wavelength with diameters of 800 nm, 400 nm, and 200 nm in 3D view (A) and 800 nm, 400 nm, 300 nm, and 200 nm in 2D view (B). (C–G) (C) Power maps of evanescent coupling between two parallel 350 nm diameter silica MNFs with overlapping length of 7.2 μm. The source is z polarized with wavelength of 633 nm. Electric field intensity distributions in x–z plane (y = 0) of (D) 5-μm and (F) 1-μm bent MNFs. The wavelength of the quasi-x polarized light is 633 nm and the diameter of the MNFs is 450 nm. The output mode profiles of (D) 5-μm and (F) 1-μm bent MNFs at the transverse crossplanes are shown in (E) and (G), respectively. The black solid lines map the topography profile of the MNFs. Reprinted with permission from Wu and Tong (Wu and Tong, 2013). Copyright 2013 Science Wise Publishing & DE GRUYTER.

For basic investigation, a straight MNF is assumed to have a circular cross-section, a smooth sidewall, a uniform diameter, and an infinite cladding with a step-index profile. By numerically solving Helmholtz equations, propagation constants (β) of guiding modes supported by the MNF can be obtained (Tong et al., 2004). Generally, when its diameter goes close to or smaller than the wavelength of the guided light, an MNF with a low-index clad (e.g., vacuum, air, or water) offers unusual properties such as tight optical confinement, high fractional evanescent fields, and tailorable waveguide dispersion, which intrigue new opportunities for manipulating light on the micro- or nanoscale. Figures 3A and 3B show power distribution (Z-direction Poynting vectors) of HE_11_ mode of silica MNFs with diameters of 800, 400, and 200 nm in 3D and 2D view, respectively. It is clear that, although an 800-nm-diameter MNF confines major energy inside the fiber, a 200-nm-diameter MNF leaves a large amount of light (>90%) guided outside as evanescent waves.

#### Evanescent Coupling between Two Parallel MNFs

Evanescent coupling between two adjacent MNFs is of special importance for designing MNF-based passive components and sensors. Usually, evanescent coupling between adjacent weakly guiding waveguides (low-index-contrast waveguides) with a certain space (e.g., a few hundred nanometers) can be described by the perturbation theory (Snyder and Love, 2012). However, when two MNFs are brought in contact, they are no longer weak coupling system, in which perturbation theory cannot be applied. Thus, we need to investigate the mode coupling by numerical calculations. Using the finite-difference time-domain (FDTD) method, Huang et al. calculated the evanescent-coupling efficiency between two air-clad parallel MNFs (Huang et al., 2007). The minimum transfer length (2.4 μm) for energy exchange is much shorter than that in weakly coupled waveguides. Also, the coupling efficiency shows an oscillating behavior depending on the overlapping length (Figure 3C). Moreover, for two MNFs with different diameter, the coupling efficiency is direction dependent: coupling light from a thinner MNF to a thicker one shows higher efficiency than in the opposite direction, which may be explained as thinner MNF have stronger evanescent field (as depicted in Figures 3A and 3B).

#### Bending Loss

Bent MNFs are important building blocks that can be readily assembled into highly compact photonic integrated circuits (PICs) or optical devices. Usually, bending losses of conventional fibers can be calculated using weakly guiding or adiabatic approximation. However, this approximation is not valid for sharply bent (a few micrometers) MNFs, which are usually high-index-contrast waveguides. Based on FDTD method, Yu et al. investigated the bending losses of MNFs with circular 90° bends, with an acceptable value of 1 dB/90° for bending radii down to micrometer level (Yu et al., 2009). As shown in Figures 3D–3G, there is virtually no power leakage for the 5-μm bent silica MNF, owing to its strong optical confinement ability. When the bending radius decreases to 1 μm, obvious energy leakage occurs around the bending region.

#### Sensing Principles

**Figure 4:**
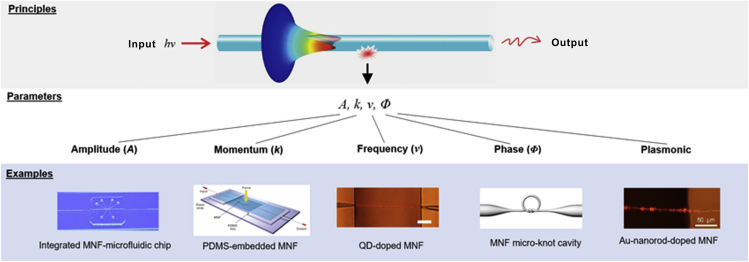
Basic Sensing Principles of MNF-Based Optical Sensors When a sample or a field to be measured interacts with light guided by an MNF, it may change the basic parameters such as amplitude (*A*), momentum (*k*), frequency, (*ν*) and/or phase (*Φ*) of the electromagnetic fields of the waveguiding mode, which can be obtained for retrieving the information of the measurand. For reference, corresponding examples of MNF-based structures are also provided beneath each parameter. In addition, plasmonic sensing is separately highlighted in contrast to photonic sensing.

Benefitting from the favorable optical properties of waveguiding MNFs, a new category of MNF optical sensors has been developed in recent years. Figure 4 schematically illustrates basic sensing principles of MNF sensors. When a sample or a field to be measured interacts with light guided by an MNF, it may change the basic parameters such as amplitude (*A*), momentum (*k*), frequency (*ν*), and/or phase (*Φ*) of the electromagnetic fields (usually electrical fields) of the waveguiding mode, which can be obtained at the output of (or sometimes via local scattering on) the MNF for retrieving the information of the measurand. For reference, corresponding examples of MNF-based structures are also provided beneath each parameter. In addition, because hybrid MNF-metal structures can convert probing photons into plasmons with high efficiency and compactness and is a promising technique for optical sensing beyond the reach of photonic approaches in some cases, it is separately highlighted as plasmonic sensing in Figure 4. In the following chapter, to be more straightforward, we introduce typical MNF-based optical sensors according to their specific structural functionalities.

### Basic MNF Structures for Optical Sensing

#### Biconical Tapered Fibers

**Figure 5:**
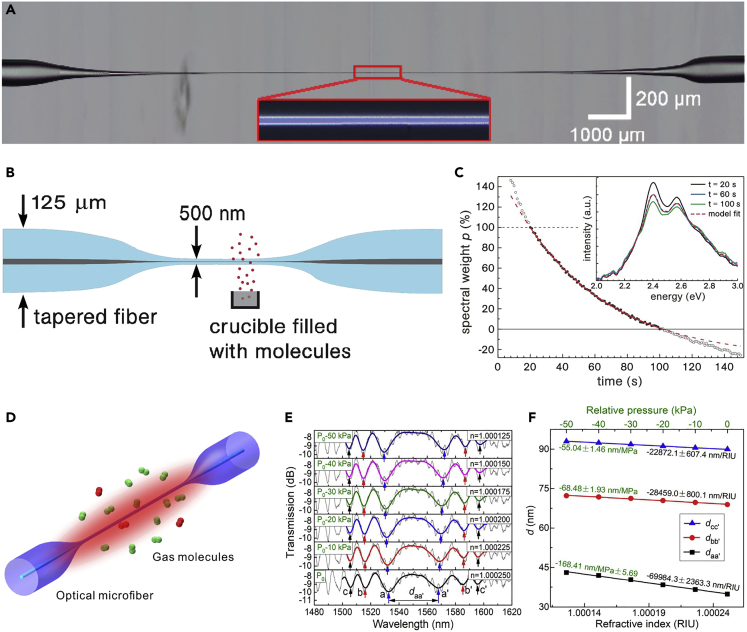
Biconical Tapered Fibers for Optical Sensing (A) The microscopic view of a tapered optical microfiber. (B) Scheme of a tapered fiber with a 500-nm diameter waist for ultrasensitive gas absorption spectroscopy. (C) Spectral investigation of the film ripening. Spectra for t = 20–100 s can be modeled as a weighted sum of the spectra at t = 20 s and t = 100 s. The dotted line extrapolates the fit for t < 20 s and t > 100 s. Reprinted with permission from Warken et al. (Warken et al., 2007). Copyright 2007 Optical Society of America. (D) The schematic illustration of the tapered optical microfiber modal interferometer-based gas sensor. (E) The variation of the interference spectrum along with decremented air pressure and surrounding refractive index. (F) The doubled sensitivities by tracing the distance between oppositely drifted twin dips. Reprinted with permission from Zhang et al. (Zhang et al., 2018). Copyright 2018 Optical Society of America.

As-drawn silica MNFs (Figure 5A), usually biconically connected to glass fibers for coupling probing light in or collecting signal light out, are the most simple and straightforward structures for optical sensing. When an MNF is used in gas or other low-index environment, in order to leave a considerably large fractional evanescent fields and generate signals large enough at its output, the diameter of the MNF is usually close to or below the wavelength of the probing light. For example, in 2007, relying on absorption of molecules adsorbed on the surface of a 500-nm-diameter silica MNF (Figure 5B), Warken et al. (Warken et al., 2007) reported an ultrasensitive molecular sensor that was possible to detect sub-monolayers of 3,4,9,10-perylene-tetracarboxylicdianhydride(PTCDA) molecules by measuring spectral absorption around 500-nm wavelength (Figure 5C). To functionalize glass MNFs with exotic materials, one of the most convenient approaches is to put them on the fiber surface. Villatoro et al. coated a 1.3-μm-diameter silica MNF with a 4-nm-thickness palladium film (Villatoro and Monzón-Hernández, 2005). Relying on its hydrogen-concentration-dependent transmission at 1,550 nm wavelength, they successfully operated the coated MNF as a fast-response (∼10 s) miniature hydrogen sensor with low detection limit. By coating a 680-nm-diameter MNF with an 80-nm-thickness gelatin layer whose RI changed with environmental humidity, Zhang et al. demonstrated an MNF optical sensor operated within a wide humidity range (9%–94% RH) with high sensitivity, good reversibility, and a 70-ms response time (Zhang et al., 2008). Despite of the fast response, the gelatin film tends to decay when the RH is higher than 95%. Moreover, surface contamination and environmental factors are likely to affect the stability of the polymer-coated MNF sensors, which were mounted in a bulky volume flow chamber. To address these issues, Cai et al. demonstrated a new functional-film-coated MNF structure for gas sensing. By using Nafion as a host polymer of crystal violet and a microflow cell as the sensing chamber, respectively, the sensor exhibits an approximately linear response to RH ranging from 30% to 100% and an estimated resolution of 0.3%RH (Cai et al., 2019).

With diameter larger than the wavelength, a biconical MNF can be operated in multimode, in which the multimode interference can be used for optical sensing (Kieu and Mansuripur, 2006). For example, Figure 5D illustrates the co-propagation of HE_11_ and HE_12_ modes in an MNF for gas sensing. The relative phase difference (*Δϕ*) of the two modes can be obtained as *Δϕ* = *Δβ·l*, where *Δβ* is the difference in propagation constants of the two modes and *l* is the interaction length. When *Δβ* and/or *l* is changed due to the environmental change (e.g., RI, temperature or strain), the relative phase change *Δϕ* will induce spectral shift at the output, leading to a loss-independent MNF sensor. It is worth noting that the RI sensitivity will reach ±∞ on either side of the dispersion turning point (DTP) due to the group effective RI difference approaching zero (Luo et al., 2015a). Zhang et al. (Zhang et al., 2018) theoretically analyze the essential conditions to achieve the DTP, and experimentally demonstrated a gas refractometer with an exceptional sensitivity as high as −69,984.3 ± 2,363.3 nm/RIU (Figures 5E and 5F). Based on numerical simulation, Sun et al. found that two DTPs exist with a decrease in the microfiber waist diameter (Sun et al., 2019). For a 1.87-μm-diameter MNF, the RI sensitivity can be as high as 95,832 nm/RIU at the second DTP, which was among the highest sensitivity reported in fiber optical RI sensors.

Recently, based on the multimode interference, a number of MNF optical sensors have been reported for measuring current (Belal et al., 2010), acceleration (Chen et al., 2011), magnetic fields (Luo et al., 2015c, Zheng et al., 2015b), strain (Li et al., 2014c, Xia et al., 2017), pressure (Yang et al., 2015), temperature (Jasim et al., 2017), and RI (Salceda-Delgado et al., 2012, Wang et al., 2011, Wang et al., 2018a). Meanwhile, by immobilizing bio-selective molecules on the surface of an MNF, highly sensitive glucose (Li et al., 2018e) and *Escherichia coli* (Li et al., 2018d) detections have also been reported.

For more flexible detection in some applications, a half biconical taper with MNF end can be used as fiber-tip-type MNF sensors (Wang et al., 2013, Ding et al., 2014). Typically, the fiber tip is fabricated by nonadiabatic fiber tapering, followed by fiber cleaving. In order to increase reflectivity, a thin layer of gold can be coated on the enface of the tip. Relying on interferometric response between the propagated and the end-reflected light, this kind of sensors has shown higher compactness and flexibility in bio/chemical detection.

#### MNF Couplers

Because the coupling efficiency is strongly dependent on the index change of fiber core or its surrounding medium, an MNF coupler is an attractive structure for highly sensitive optical sensing (Jung et al., 2009, Liu et al., 2019a). So far, MNF couplers have been widely employed for detection of microforce (Chen et al., 2014), magnetic fields (Luo et al., 2015b), current (Yan et al., 2015) seawater salinity (Wang et al., 2015c), temperature (Jiang et al., 2018, Ming et al., 2012, Zhao et al., 2018), and humidity (Bai et al., 2019, Peng et al., 2019, Zhao et al., 2019).

**Figure 6:**
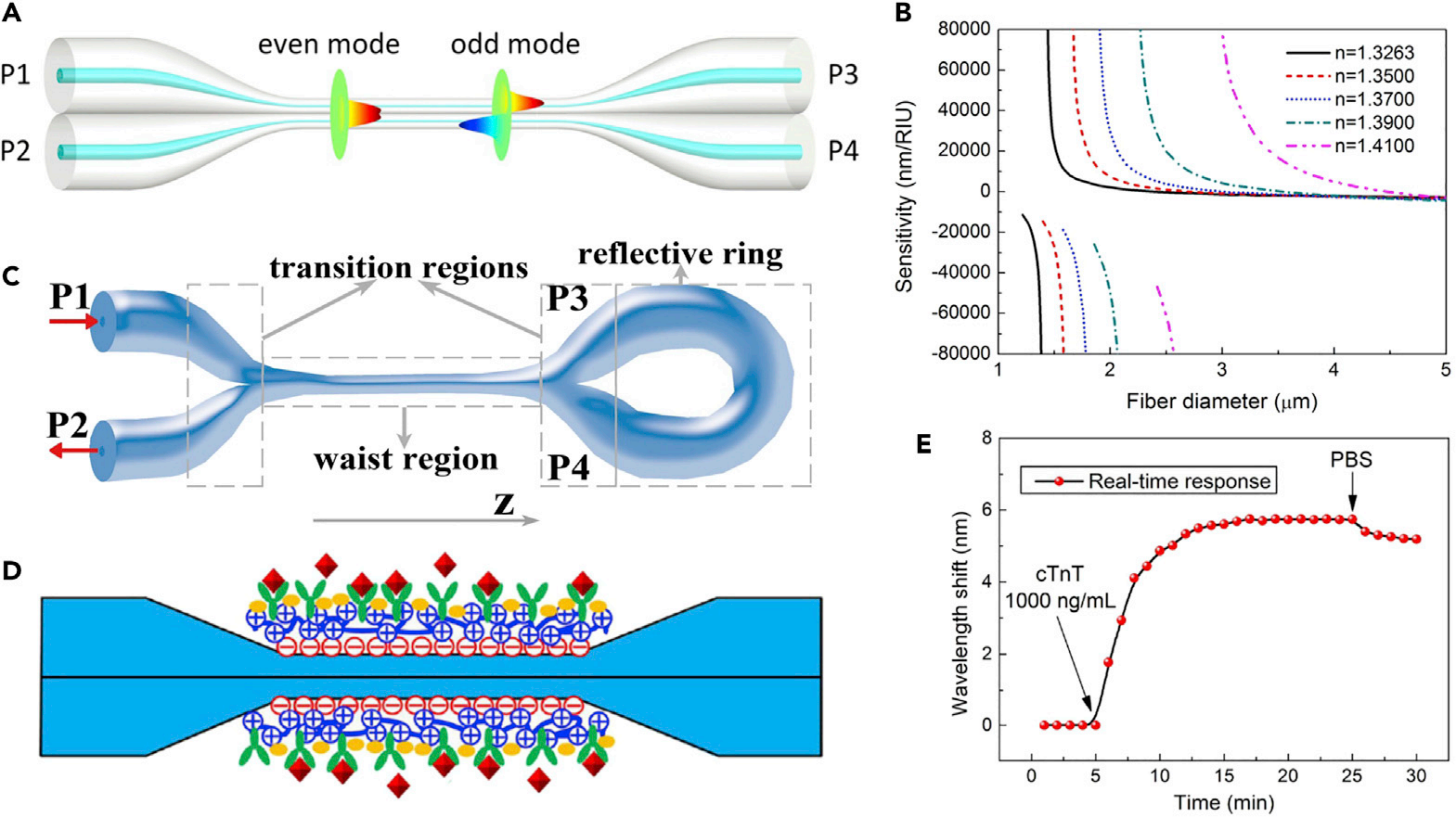
MNF-Based Mach-Zehnder Interferometers for Optical Sensing (A) Schematic diagram of a microfiber coupler. (B) Calculated RI sensitivities as a function of fiber diameter ranging from 1 to 5 μm with different ambient RIs at wavelength 1,550 nm. Reprinted with permission from Li et al. (Li et al., 2016). Copyright 2016 AIP Publishing. (C) Schematic of the reflective tapered fiber coupler with Sagnac loop structure. Reprinted with permission from Pu et al. (Pu et al., 2016). Copyright 2016 IEEE. (D) Schematic diagram of fiber surface modification and antibody immobilization. (E) The real-time response curve of the sensor to cTnT molecules (1,000 ng/mL). Reprinted with permission from Li et al. (Li et al., 2018c). Copyright 2018 Elsevier (B)V.

Note that when an optical microfiber coupler (Figure 6A) works near the turning point of effective group index difference between the even supermode and odd supermode, high refractive index (RI) sensitivity can be achieved (Figure 6B) (Li et al., 2016). In addition to the parallel structure (Figure 6A), Sagnac loop structure (Figure 6C) can enhance the reflection of microfiber coupler, which is promising for assembling MNF sensors with low loss, compactness, low cost, and ultrahigh sensitivity (Pu et al., 2016, Zu et al., 2019). Relying on birefringence-induced Vernier effect in optical fiber modal interferometers, Li et al. demonstrated the sensitivity enhancement of a microfiber coupler for refractive index (RI) sensing, achieving an ultrahigh sensitivity of 35,823.3 nm/RIU using a microfiber coupler with a width of 3.2 μm (Li et al., 2018c). When the surface of the microfiber coupler was immobilized antibody (Figure 6D), human cardiac troponin was realized with a limit of detection of 1 ng/mL (Figure 6E).

#### Mach-Zehnder Interferometers

**Figure 7:**
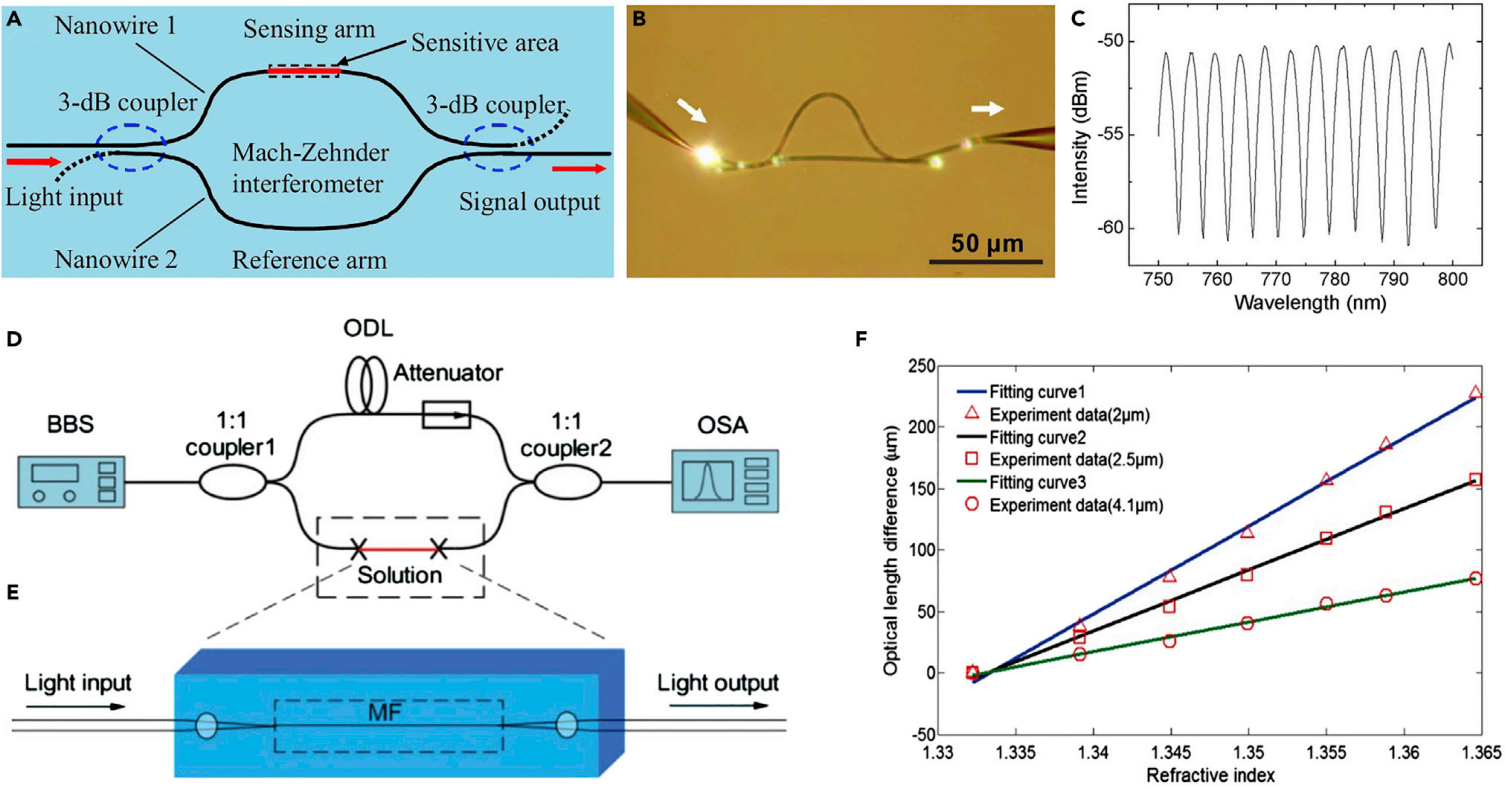
MNF-Based Mach-Zehnder Interferometers for Optical Sensing (A) Schematic diagram of a proposed MNF sensor with a Mach-Zehnder interferometer. Reprinted with permission from Lou et al. (Lou et al., 2005). Copyright 2005 Optical Society of America. (B) Optical microscope image of an MNF Mach-Zehnder interferometer assembled with two 480 nm diameter tellurite MNFs. (C) Typical transmission spectrum of an MNF Mach-Zehnder interferometer. Reprinted with permission from Li et al. (Li and Tong, 2008). Copyright 2008 Optical Society of America. (D and E) (D) Schematic configuration of a Mach-Zehnder interferometer-based RI sensor; (E) schematic diagram of the sensing arm. BBS, broadband light source; ODL, optical delay line; OSA, optical spectrum analyzer; MF, microfiber. (F) Optical length variation with the RI at different microfiber diameters. Reprinted with permission from Wo et al. (Wo et al., 2012). Copyright 2012 Optical Society of America.

MNF-based Mach-Zehnder interferometer is one of the mostly used structures for optical MNF sensing. Based on numerical simulation, an MNF-based Mach-Zehnder interferometer (Figure 7A) can provide a sensitivity one order of magnitude higher than those of conventional waveguide Mach-Zehnder interferometers (Lou et al., 2005). Experimentally, the MNF-based Mach-Zehnder interferometers, with dimensions of tens to hundreds of micrometers (Figure 7B), show good interference fringes with extinction ratios of 10 dB (Figure 7C). Therefore, incorporating optical MNFs into Mach-Zehnder interferometers for phase-sensitive optical measurement may offer high sensitivity with small footprint. To date, a number of MNF-based Mach-Zehnder interferometers have been employed for phase-sensitive optical sensing. For example, by using a 2-μm-diameter silica MNF as the sensing arm, and using a tunable optical delay line to compensate the change of the optical length difference (Figures 7D and 7E), Wo et al. (Wo et al., 2012) reported a simple and robust RI sensor with an RI sensitivity as high as 7,159 μm/RIU (Figure 7F). Relying on near-field coupling, Li et al. reported a hybrid photon-plasmon Mach-Zehnder interferometer by integrating Ag nanowires with MNFs, realizing a photon-to-plasmon conversion efficiency up to 92%. When the plasmonic probe was exposed to NH_3_, the group index of the Ag nanowire was changed due to the change of the resistance, resulting in spectral shifts of the interference peaks. The detection limit was much lower than 80 ppm, with a fast-response time of about 400 ms (rising time) and 300 ms (falling time), which is on the same order of other types of fast-response gas sensors (Gu et al., 2008).

Recently, a PDMS-microfiber-based Mach-Zehnder interferometer (Martincek and Kacik, 2018), a PDMS-assisted hybrid microfiber Mach-Zehnder interferometer-knot resonator structure (Liu et al., 2019c), and optimized microfiber-based Mach-Zehnder interferometers (Liu et al., 2019b, Su and Zhou, 2018, Yaghobi and Karimi-Alavijeh, 2019) have been reported for highly sensitive measurement of RI, nanometer displacements, and bending.

#### MNF Gratings

**Figure 8:**
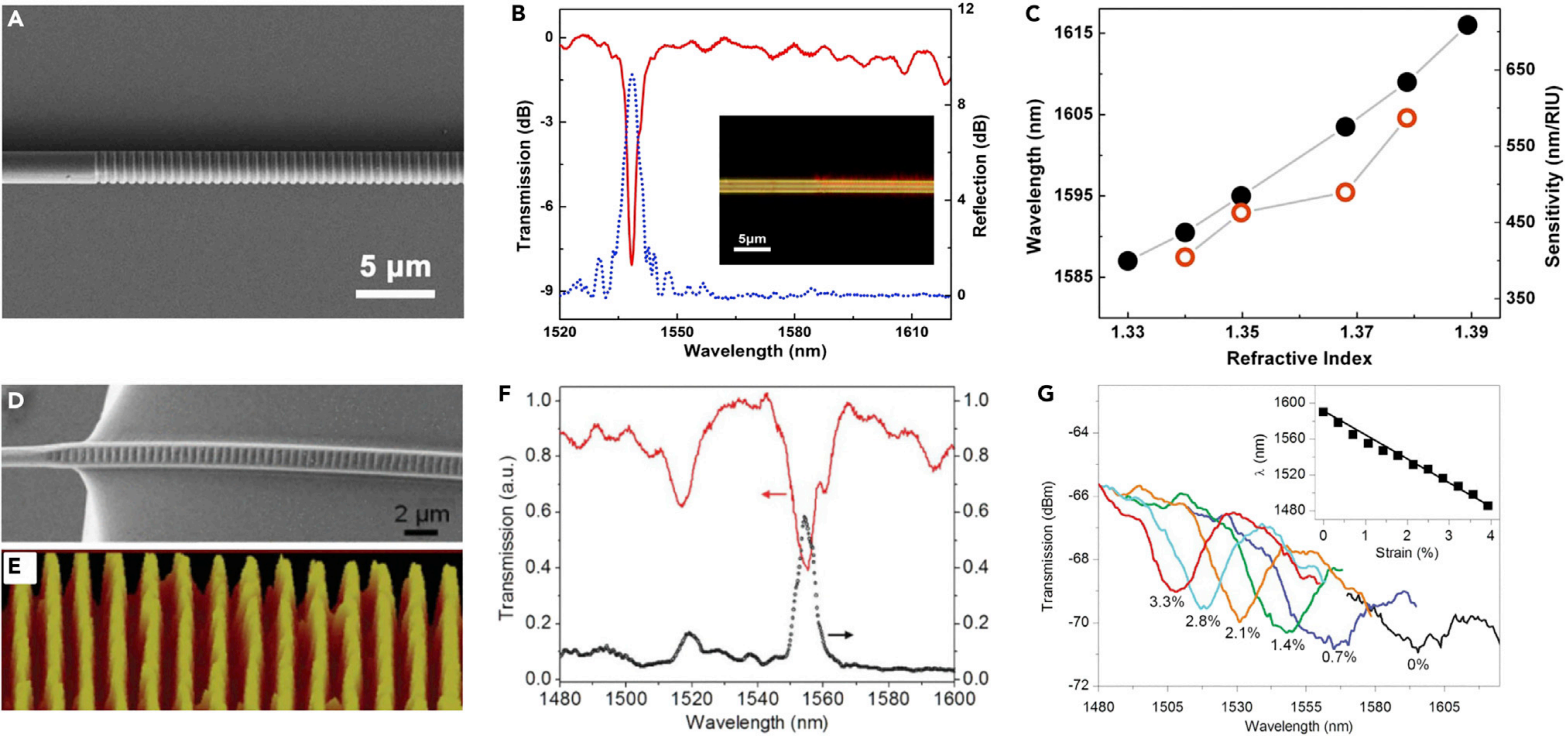
MNF-Based Gratings for Optical Sensing (A) SEM image of an MNFBG inscribed on a 1.8-μm-diameter silica MNF. (B) Transmission and reflection spectra of the MNFBG. Inset: optical microscope image of the MF and MFBG guiding a 633 nm light. (C) Dependence of the reflection wavelength shift on the ambient RI (black dot line) and the corresponding RI sensitivity (red hollow dot line) of the MNFBG used for measuring the RI of a glycerin solution. Reprinted with permission from Liu et al. (Liu et al., 2011b). Copyright 2011 Optical Society of America. (D) SEM image of the grating area of the imprinted MNFBG from a 1.3-μm-diameter MNF. (E) AFM image of the grating mold used for imprinting a grating segment of a 1.2-μm-wide MNFBG. (F) Transmission and reflection spectra of the a typical imprinted MNFBG (2.3-μm wide by 1.4-μm thick). (G) Transmission spectra of an MNFBG (2.5-μm wide by 1.4-μm thick) with tensile strain increasing from 0% to ~4%. Inset: Bragg wavelength shift of the 1590-nm dip under tensile strain from 0% to ~4%. Reprinted with permission from Gu et al. (Gu et al., 2013a). Copyright 2012 IEEE.

Fiber Bragg gratings (FBG) is one of the most successful fiber-based structures for optical sensing (Rao, 1997). Owing to its high compactness, strong near-field interaction with the surrounding medium, and high resistance to mechanical and thermal shocks, MNF gratings may offer special advantages in optical sensing including high sensitivity, small footprint, large dynamic range, and fast response and have been attracting increasing interest in recent years (Kou et al., 2012). To fabricate an MNF Bragg gratings (MNFBG) with much smaller fiber diameter and shorter overall length, a great number of techniques, including femtosecond laser pulse irradiation (Fang et al., 2010, Liao et al., 2019), 193 nm ArF excimer laser inscription (Ran et al., 2011), and focused ion beam (FIB) milling (Kou et al., 2011, Liu et al., 2011b, Nayak et al., 2011), have been reported. Figure 8A shows a 518-μm-length 1.8-μm-diameter MNFBG fabricated by FIB. A reflection peak occurs at the wavelength of 1,538.5 nm, which agrees well with the transmission dip located at 1,538.4 nm (Figure 8B). When the MNFBG was used for RI sensing, the transmission dip shows an evident monotonous redshift from 1.587 μm to 1.616 μm, achieving a sensitivity of 660 nm/RIU for RI measurement (Figure 8C). Besides the above-mentioned methods, nanoimprint technique has also been used for fabricating polymer MNFBG. For example, by using standard plane reflection grating as a mold, Gu et al. (Gu et al., 2013a) fabricated PMMA MNFBG with grating period of ∼551 nm and grating depth >10 dB around 1.5-μm wavelength (Figures 8D and 8E). Owing to its stretchability, the PMMA MNFBG was used for strain sensing with a sensitivity as high as −2.5 pm/με (Figure 8F). Recently, based on MNFBGs functionalized with bio-recognition molecules, *in-situ* DNA hybridization and label-free cardiac biomarker detection have been reported (Liu et al., 2018). By using a polymer-film-coated MNFBG, Zhang et al. demonstrated an RH sensor with relatively fast response, large measurement range, and high stability (Zhang et al., 2019a). To overcome the temperature cross-sensitivity of the MNFBG refractometer, Ran et al. reported a novel refractive-index-temperature dual-sensing paradigm involving the third harmonic Bragg resonance (Ran et al., 2019).

In addition to the above-mentioned MNFBGs, a variety of designs including long-period gratings (LPG) (Fan et al., 2016, Li et al., 2017a, Xu et al., 2016, Xuan et al., 2009, Xuan et al., 2010a, Zhang et al., 2014), evanescently coupled gratings (Xu et al., 2009, Xu et al., 2010), type IIa Bragg gratings (Ran et al., 2015), and chirped Bragg gratings (Xiao et al., 2016) have also been fabricated on MNFs and used for high-sensitivity optical sensing.

#### MNF Resonators

**Figure 9:**
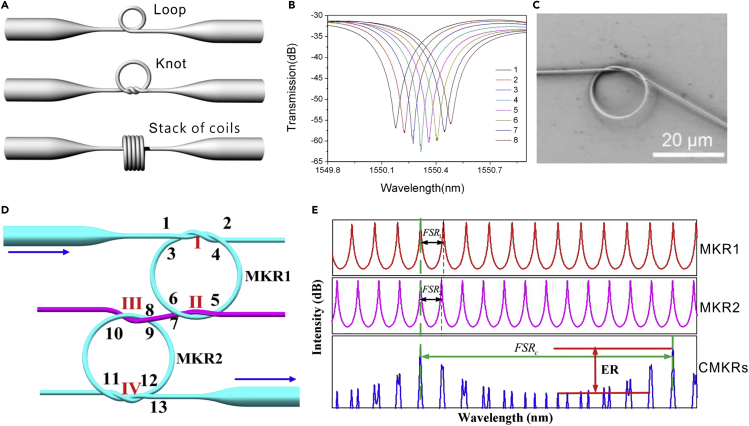
MNF-Based Resonators for Optical Sensing (A) Schematic diagram of typical MNF-based optical resonators in forms of loop, knot, and stack of coils. (B) Spectral shifts of a resonant peak caused by index change of the solution. The eight peaks are obtained by adding a 5-μL ethanol into a 500-μL water in steps. Reprinted with permission from Guo and Tong (Guo and Tong, 2008). Copyright 2008 Optical Society of America. (C) SEM image of an MNF knot resonator. (D) Schematic diagram of CMKRs. (E) Transmission spectra of (1) MKR1, (2) MKR2, and (3) CMKRs. Reprinted with permission from Wu et al. (Xu et al., 2015). Copyright 2015 Optical Society of America.

When an MNF is assembled into a closed loop, a ring resonator can be formed by evanescent coupling at the overlapping area. Typical geometries (e.g., loosely assembled loop, tied knot, and a stacked multicoil) of this kind of resonator are schematically illustrated in Figure 9A. For reference, Figure 9B shows an SEM image of an MNF knot resonator. Depending on the ring size and geometry, typical Q-factor of an MNF resonator varies from 10^2^ to 10^6^ (Tong and Sumetsky, 2011). As the simplest structure in MNF resonators, the MNF loop resonator has been intensively investigated for temperature (Sumetsky et al., 2006) and RI (Shi et al., 2007, Sun et al., 2012, Wang et al., 2012b) sensing. Although the MNF loop is simple and high-Q, the loop structure maintained by the van der Waals and electrostatic forces is difficult to operate with high mechanical stability, especially in liquids. To enhance the robustness, Guo et al. reported a copper-rod-supported loop resonator assembled by wrapping a 2.8-μm-diameter MNF around a 460-μm-diameter copper rod (Guo et al., 2007, Guo and Tong, 2008); under critical coupling condition, the resonance peaks shift to longer wavelength with the increase of the RI (Figure 9B), with a sensitivity of 1.8×10^−5^ for RI measurement.

To enhance the mechanical stability of free-standing MNF ring resonators without substrates, Jiang et al. tied a free-standing MNF into a knot (Jiang et al., 2006a). As shown in Figure 8C, the knot structure was maintained by the friction of the microfiber at the joint area under the tension of the elastically bent knot and was proved highly stable in water. Based on MNF knot resonators, a variety of structures have been reported for temperature (Li et al., 2017b, Wu et al., 2009, Wu et al., 2012), humidity (Wu et al., 2011), magnetic field (Li and Ding, 2012), and electric field (Hou et al., 2017) sensing. Benefitted from the high Q-factor and the miniaturized structure, the abovementioned sensors offered high sensitivity and fast response. Firstly demonstrated by Sumetsky in 2004 (Sumetsky et al., 2004), the multicoil resonators have also been widely explored for measurement of RI (Xu and Brambilla, 2007, Xu and Brambilla, 2008, Xu et al., 2007, Yin et al., 2018), acoustic waves (Chen et al., 2012), current (Xie et al., 2014), and in-line absorption (Lorenzi et al., 2011). Based on cascaded microfiber knot resonators (CMKRs) with spectrum magnification function of Vernier effect (Figures 8D and 8E), the CMKRs shows high sensitivity of 6,523 nm/RIU and detection resolution up to 1.533 × 10^−7^ RIU (Xu et al., 2015), which can be widely used for chemical and biological sensing. More recently, a novel design of nested optical fiber based on multiple microfiber knot resonators was demonstrated. The periodic spectrum of this device may find applications from optical sensing to communications (Yi et al., 2019).

### Hybrid MNF Structures for Advanced Optical Sensing

#### Functionalized Polymer MNF Sensors

**Figure 10:**
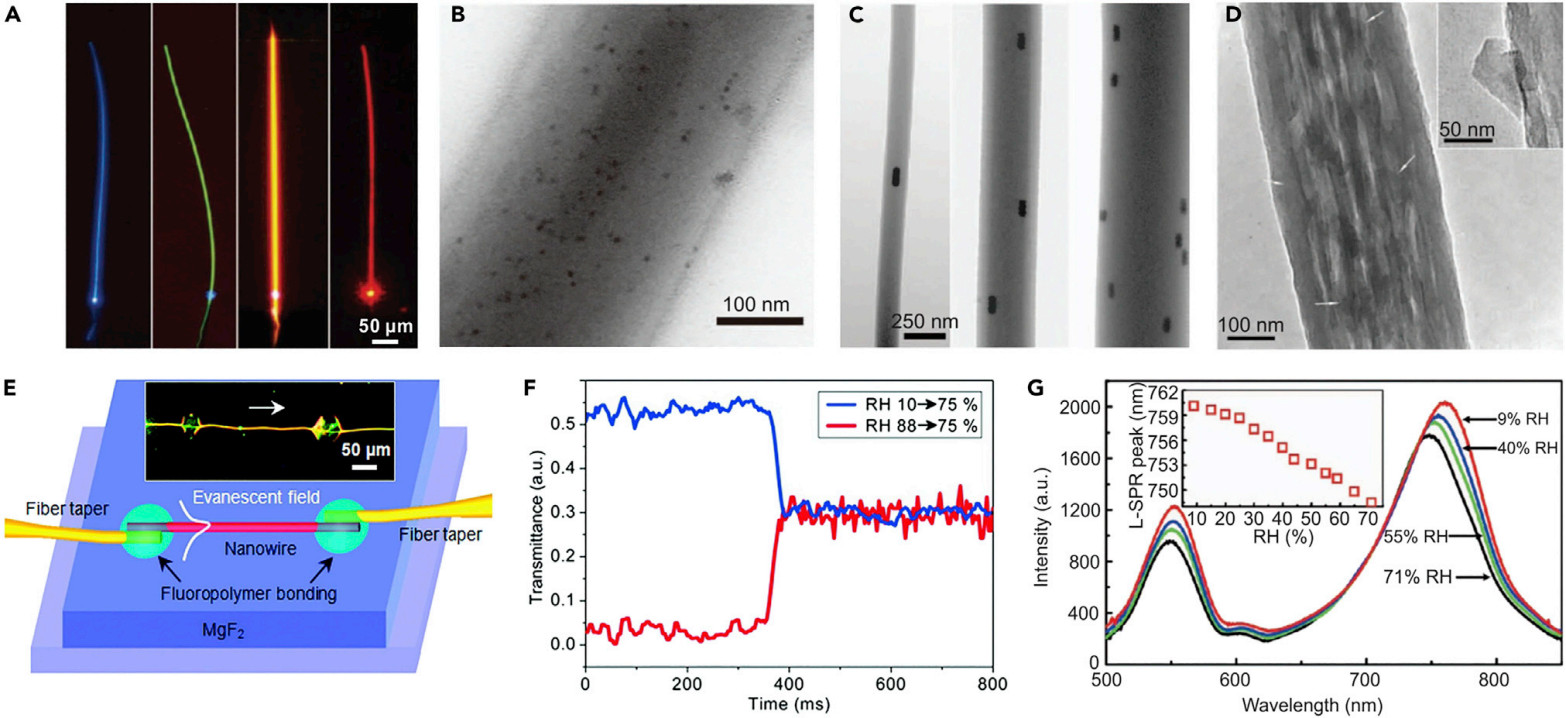
Functionalized Polymer MNFs for Optical Sensing (A) Typical light-emitting polymer nanofibers excited by 355-nm light. The nanofibers are doped with different fluorescent dyes to emit different colors of light. Reproduced with permission from Gu et al. (Gu et al., 2010). Copyright 2010 American Chemical Society. (B) A 280-nm-diameter PS nanofiber doped with CdSe quantum dots. Reproduced with permission from Meng et al. (Meng et al., 2011). Copyright 2011 John Wiley & Sons, Inc. (C) Three PAM nanofibers doped with aligned GNRs. Reproduced with permission from Wang et al. (Wang et al., 2012a). Copyright 2012 American Chemical Society. (D) High-magnification TEM image of a graphene-doped poly(vinyl acetate) nanofiber. The inset shows an enlarged image of graphene embedded in the sidewall of a poly(vinylacetate) nanofiber. Reproduced with permission from Bao et al. (Bao et al., 2010). Copyright 2010 John Wiley & Sons, Inc. (E) Schematic illustration of a single polymer nanowire sensor. Inset: optical microscope image of a MgF_2_-supported 410-nm-diameter PAM nanowire with a 532-nm-wavelength light launched from left side. Reprinted with permission from Gu et al. (Gu et al., 2008). Copyright 2008 Optical Society of America. (F) Typical time-dependent transmittance of the sensor reveals the response time of about 24 ms when RH jumps from 10% to 75% and 30 ms when RH falls from 88% to 75%. (G) Scattering spectra of the GNR exposed to air of varying RH. Inset: the dependence of LSPR peak on the RH of ambient air. Reprinted with permission from Wang et al. (Wang et al., 2012a). Copyright 2012 American Chemical Society.

In addition to geometric structures, an MNF can also be functionalized by adding functional materials into the fiber core. Because the functional materials for optical sensing, e.g., organic molecules, typically cannot suffer the high temperature of the molten glass or polymer, MNFs drawn from room temperature polymer solutions become the optimum substrates to host the functional materials. As shown in Figures 10A–10D, polymer MNFs are excellent hosts for exotic dopants or inclusions, including dye molecules (Gu et al., 2010), chemical indicators (Gu et al., 2008, Gu et al., 2009), quantum dots (Meng et al., 2011), metal nanoparticles (Wang et al., 2012a), and graphene (Bao et al., 2010). Unlike biconical silica MNFs drawn from standard optical fibers, optical waveguiding in a single polymer MNF was realized by using evanescent coupling technique as illustrated in Figure 10E. Owing to the small diameter and large surface-to-volume ratio of the polymer MNF that enable rapid diffusion or evaporation of the gas molecules, a single-polymer MNF sensor achieved remarkably fast response. For example, the response time of a polymer MNF humidity senor is about 24 ms (Figure 10F), which is one or two orders of magnitude faster than those of existing RH sensors (Peng et al., 2018). Benefitted from the immunity to photobleaching, Wang et al. demonstrated a low-power fast-response optical humidity sensor by embedding a 540-nm-diameter PAM MNF with Au nanorods whose localized surface plasmonic resonance (LSPR) frequency was strongly dependent on environmental RI. As shown in Figure 10G, the scattering intensity of the GNR decreased monotonically with increasing RH, offering a sensitivity of ∼0.07 dB/% RH and an estimated resolution better than 1% RH (Wang et al., 2012a). Recently, more functional materials, such as proteins (Sun et al., 2015), ZnO nanostructures (Irawati et al., 2017a), CdSe quantum dot (Irawati et al., 2017b), silver nanoparticles (Shahzad et al., 2019), and molecular imprinted nanoparticles (Shrivastav et al., 2019) have been incorporated with MNFs for optical sensing.

#### Plasmonic-Nanostructure-Activated Microfiber Sensors

Metallic nanostructures (e.g., nanoparticles, nanowires) possess plasmonic resonances that spatially confine light on the nanometer scale. In the ultimate limit of a single nanostructure, the electromagnetic field can be strongly concentrated in a volume of only a few hundreds of nanometer or less. This optical nanofocus has been intensively explored for plasmonic sensing (Gu et al., 2013b, Gu et al., 2014, Liu et al., 2011a, Monzon-Hernandez et al., 2010). It is worth noting that the peak extinction cross-section of a metallic nanostructure is comparable with the mode area of an MNF nanofiber. When the light is guided through an MNF, the guiding modes maintain their small mode areas all the way along the entire length of the MNF, enabling strong interaction between the light and the metallic nanostructures and making it possible to transfer light to LSPR in metallic nanoparticles with high efficiency. In addition, using ultracompact near-field interaction, direct coupling of plasmonic nanowires with optical MNF is an effective approach to assemble hybrid nanophotonic components and sensors (Guo et al., 2009). In 2015, by using PdAu nanowires as plasmonic waveguides, Gu et al. reported a hybrid photon–plasmon Mach-Zehnder interferometer-based hydrogen sensor that was capable of detecting low concentrations of hydrogen (up to 10%) at room temperature with response times on the order of seconds much faster than many other hydrogen sensors that exploit phenomena at the nanoscale (Gu et al., 2015a).

**Figure 11:**
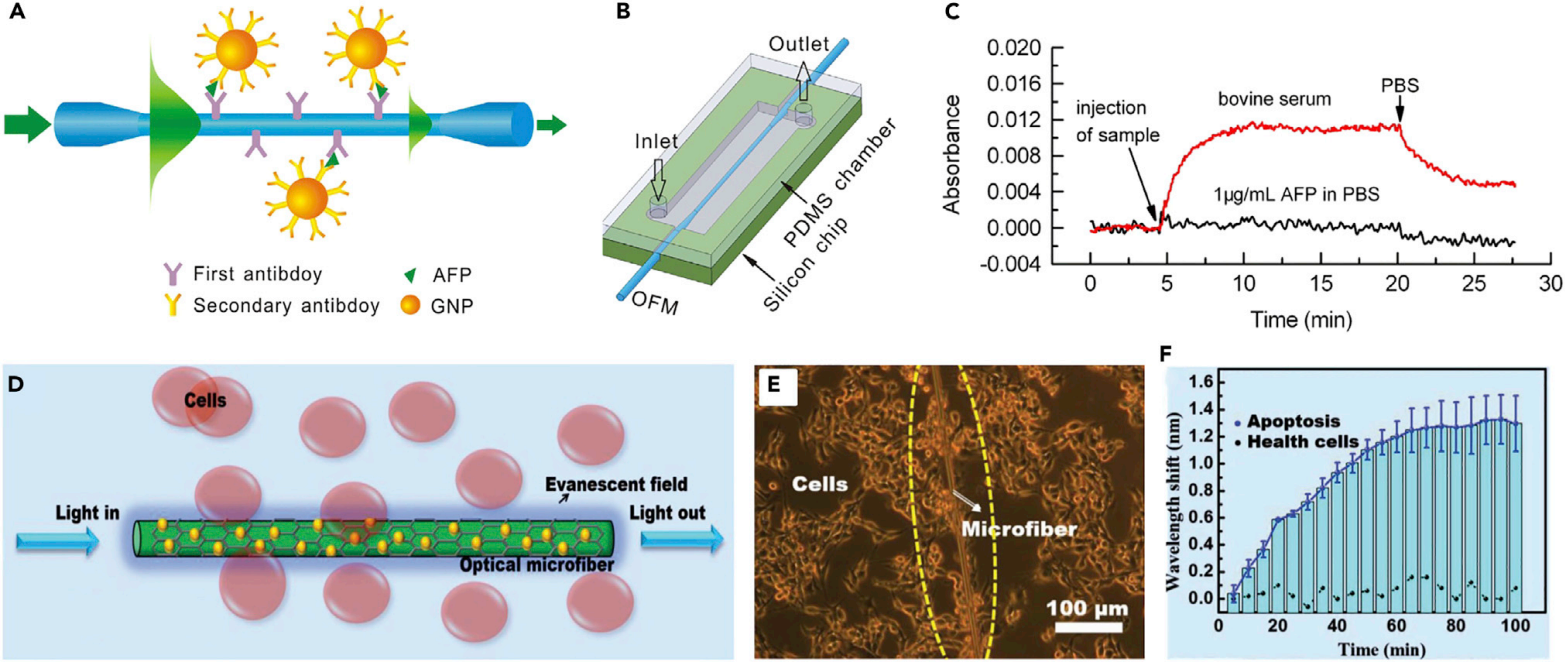
Plasmonic-Nanoparticle-Activated Microfiber Sensors Schematic diagram of the immunoassay for α-Fetoprotein (AFP) detection using GNPs as signal amplification labels. (B) Schematic diagram of an OFM sensor device with an integrated PDMS chamber for sample delivery. (C) Absorbance responses of the OMF sensor to 1 μg/mL AFP in PBS and bovine serum without AFP, respectively. Reprinted with permission from Li et al. (Li et al., 2014a). Copyright 2013 Elsevier B.V. (D) Schematic of the process of detecting cyt c by an optical microfiber. (E and F) (E) Optical microscopy image and (F) measured wavelength shift with H_2_O_2_ (blue solid line) and without H_2_O_2_ (black dotted line) of cyt c monitoring in cells in the apoptosis tracing. Reprinted with permission from Li et al. (Li et al., 2018a). Copyright 2018 John Wiley & Sons, Inc.

**Figure 12:**
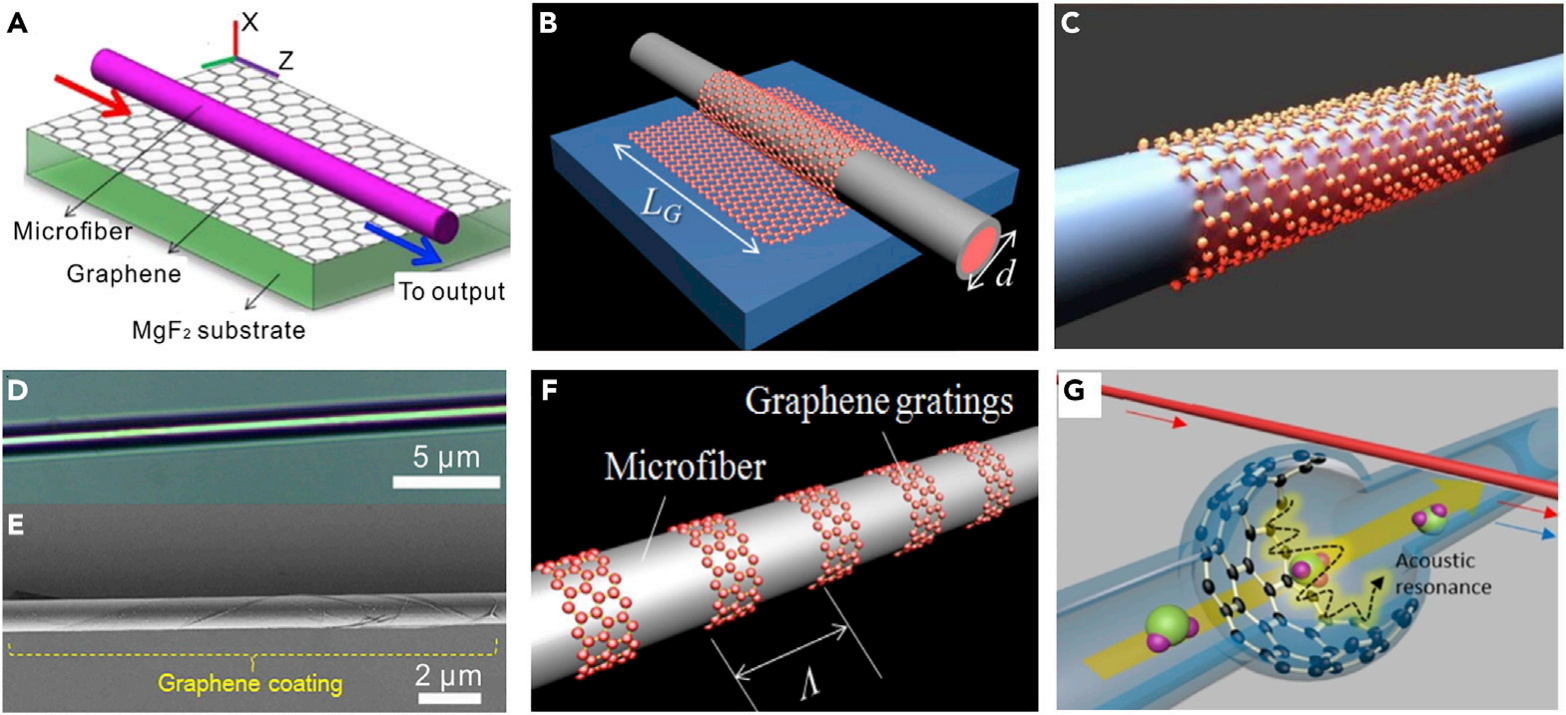
Typical Configurations of Hybrid Graphene-MNF Structures (A and B) The graphene layer is placed (A) beneath (Reprinted with permission from Yao et al. (Yao et al., 2013). Copyright 2013 Optical Society of America) or (B) on the top of a substrate-supported MNF (Reprinted with permission from Yao et al. (Yao et al., 2014a). Copyright 2014 Optical Society of America.) (C) Wrapped around a free-standing MNF. (D) Optical microscope images of the GCM area. (E) SEM image of the GCM. Reprinted with permission from Li et al. (Li et al., 2014b). Copyright 2014 American Chemical Society. (F) Schematic of a graphene grating. Reprinted with permission from Yao et al. (Yao et al., 2014b). Copyright 2014 Optical Society of America. (G) Schematic of the GBMR. A rGO film is incorporated in a bottle-shaped silica capillary resonator, and a pump laser is coupled in using a tapered fiber. Reprinted with permission from Yao et al. (Yao et al., 2017). Copyright 2017 American Chemical Society.

Besides hydrogen sensing, gold-nanoparticles-decorated silica MNF enables a new platform for biosensing. For example, using gold nanoparticles (GNPs) as amplification labels (Figures 11A), Li et al. reported a sensitive and selective cancer biomarker sensor (Li et al., 2014a). Enabled by the microfluidic channel (Figure 12B), one can monitor the immunoassay in real time, and the sensor can be reused for at least 10 cycles without significant losses in sensitivity. Recently, relying on an interfacial sensitization effect coupled with the plasmonic electromagnetic enhancement of silver nanoparticles and chemical enhancement of graphene platforms, Li et al. demonstrated an optical microfiber aptasensor (Figures 11D–11F) with the lowest limit of detection of 6.82 × 10^−17^ M, which is approximately five orders of magnitude lower than those of existing methods (Li et al., 2018a).

It is worth noting that reduction of plasmon resonance line width is an effective way to enhance the sensitivity. By coupling a gold nanorod to a whispering gallery cavity of a silica microfiber, Wang et al. obtained a single-band 2-nm-line-width plasmon resonance in an Au nanorod around a 655-nm-wavelength. The significantly reduced LSPR line width may open new opportunities for pushing the limits of plasmon-based techniques (Wang et al., 2015b), e.g., increasing the sensitivity of LSPR-based sensors to the same level of the propagation surface plasmon sensing systems (Tong, 2018). By taking advantage of spatially localized single-band narrow plasmon resonance of a nanoparticle coupled to a whispering gallery cavity mode of a microfiber, a hydrogen gas detection with enhanced sensitivity (Gu et al., 2015b) and a sensitive humidity sensor with a 1.5-mm spatial resolution (Zhou et al., 2019) are realized.

#### Graphene Functionalized Microfiber Sensors

Graphene is a unique two-dimensional material composed of carbon in a honeycomb lattice with atomic thickness and has spurred remarkable advances ranging from chemical physics and materials science, to optoelectronics, mechanics, and thermal processes (Wu et al., 2018). Using graphene atomic layers to detect adsorbed gas molecules has been attracting much attention due to its high sensitivity and low detection limit (Schedin et al., 2007). Recently, graphene-decorated MNFs become a new platform for optical gas sensing, with typical structures shown in Figure 12. The graphene layer can be placed either beneath (Figure 12A) or on the top (Figure 12B) of a substrate-supported MNF, wrapped around a free-standing MNF (Figure 12C). Figures 12D and 12E show a typical micrograph and an SEM image of bilayer-graphene-clad microfiber, respectively. Based on graphene-decorated MNFs, Yao et al. has reported a series of optical sensors for high-sensitivity gas sensing, including a multimode-interferometer NH_3_ gas sensor (Yao et al., 2014a), a graphene-MNFBG (Figure 12F) gas sensor (Yao et al., 2014b), and a microfiber interrogated whispering gallery mode optomechanical gas sensor (Figure 12G) with an unprecedented high sensitivity (1 ppb) for NH_3_ gas detection (Yao et al., 2017). Meanwhile, using hybrid graphene-MNF structures for current (Zheng et al., 2015a), temperature (Sun et al., 2016, Wang et al., 2018b), humidity (Azzuhri et al., 2018), magnesium (Yasin et al., 2018), and DNA (Huang et al., 2017) sensing have also been reported by other groups. Additionally, many other 2D materials, such as WS_2_ (Chen et al., 2018b), SnS_2_ (Lu et al., 2018), and black phosphorus (Yin et al., 2019) have recently been successfully incorporated with MNFs for all light control and optical sensing, which may bestow more versatilities to MNFs for optical sensing applications.

#### Optofluidic Microfiber Sensors

**Figure 13:**
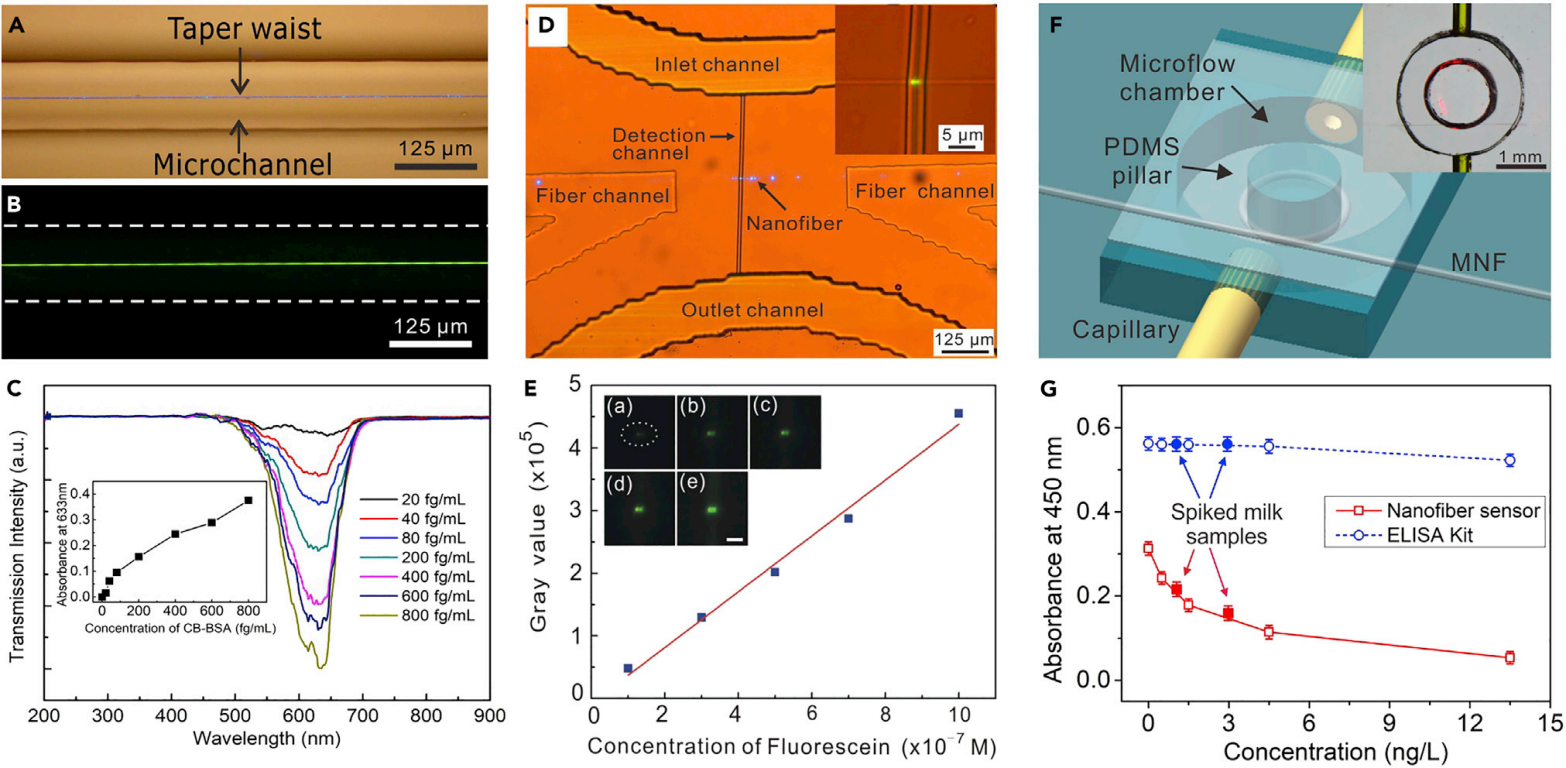
Microfluidic Chip Integrated MNF Optical Sensors (A) Optical micrograph of a 1.5-μm-diameter MNF guiding 473-nm-wavelength laser integrated into a microfluidic channel. (B) Optical micrograph of the fluorescence excited by evanescent field outside the MNF. (C) Transmission spectra of different CB-BSA concentrations for a 900-nm-diameter MNF. Inset: absorbance at 633-nm-wavelength versus BSA concentrations. Reprinted with permission from Zhang et al. (Zhang et al., 2011). Copyright 2011 of the Royal Society of Chemistry. (D) Optical micrograph of a nanofiber-microfluidic chip with a nanofiber crossed a detection channel. Inset: optical micrograph of an 800-nm-diameter nanofiber crossed a 5-μm-wide channel. (E) Calculated fluorescence intensity versus the concentration of fluorescein. Insets: (A–E) optical micrographs of the fluorescence spots excited by evanescent field outside an 800-nm-diameter nanofiber with fluorescein concentrations of 1 × 10^−7^, 3 × 10^−7^, 5 × 10^−7^, 7 × 10^−7^, and 1 × 10^−6^ M, respectively. Reprinted with permission from Zhang et al. (Zhang et al., 2015). Copyright 2015 Optical Society of America. (F) Schematic illustration of a coiled nanofiber sensor. Inset: a micrograph of a coiled nanofiber sensor. (G) Peak absorbance at 450 nm versus concentration of CAP solutions, recorded by the nanofiber sensor (red square) and standard ELISA analysis (blue circle), respectively. Reprinted with permission from Mei et al. (Mei et al., 2019). Copyright 2015 American Chemical Society.

Note that most of the abovementioned MNF sensors used MNFs suspended in air or mounted in a bulky volume flow chamber, thus surface contamination and environmental factors are likely to affect the stability of these sensors. Integrated MNFs with microfluidic chips is an effective way to enhance the stability of the MNF sensors and achieve high sensitivity because the pronounced evanescent field of an MNF can directly interact with samples. Moreover, by taking the advantages of the network microchannels, the MNF surface can be renewed after each measurement, which is critical for a biosensor. For example, Zhang et al. reported integrated nanofiber-microfluidic devices for ultrasensitive absorption (Zhang et al., 2011) and fluorescence measurements (Li et al., 2015) by enclosing optical nanofibers into microchannels with a detection length of 2.5 cm (Figures 13A and 13B). When the sensor was applied for the BSA measurement (Figure 13C), the detection limit was down to 10 fg/mL, and the sensitivity is several orders of magnitude higher than that of standard and modified Bradford assay methods. By taking the advantages of the tightly confined large fractional evanescent fields of the waveguiding nanofiber, as well as the short detection length defined by the width of a narrow microfluidic channel (Figures 13D and 13E), Zhang et al. reported a femtoliter-scale optical nanofiber sensor, providing a compact and versatile sensing platform for sensitive and fast detection of ultralow-volume samples, as well as studying the dynamics of single molecule (Zhang et al., 2015). Recently, by using a PDMS-pillar-supported structure (Figure 13F), Mei et al. developed a robust coiled optical nanofiber sensor for highly sensitive detection of chloramphenicol (Figure 13G), achieving a detection limit down to 0.5 ng/L (Mei et al., 2019). Besides the microfluidic chips with networks of microchannels, capillary (White et al., 2006) or photonic microcell (Jin et al., 2014) has been successfully integrated with MNF for single nanoparticle (Yu et al., 2014), lead ion (Yap et al., 2018), and RNA (Liang et al., 2017) sensing. For reference, to compare the state-of-the-art performance of different types of hybrid MNF sensors, we summarize configuration, measurand, sensitivity, limit of detection (LOD), and resolution of the recently reported works in Table 1.

**Table 1:** Comparison of the State-of-the-Art Performance of Hybrid MNF Structures for Optical Sensing

Type of Hybrid MNF Sensors	Configuration	Measurand	Sensitivity	LOD/Resolution	Reference
Functionalized polymer MNF sensors	Protein (avidin/BSA) nanowire	Biotin	/	0.2ppb	Sun et al., 2015
	PMMA microfiber loop coated with ZnO	RH	0.1746 dBm/%	6.17%	Irawati et al., 2017a
	CdSe quantum dot doped MMA microfiber	Temperature	58.5 pm/°C	0.292°C	Irawati et al., 2017b
	UCNPs with PMMA and silver microfiber	Temperature	0.0095 K^−1^	/	Shahzad et al., 2019
Plasmonic nanostructure activated microfiber sensors	Gold nanoparticles	Alpha-fetoprotein	/	2ng/mL in bovine serum	Li et al., 2014a
	Silver-decorated graphene	Cytochrome *c*	0.583 nm/logM	6.82 × 10^−17^ M	Li et al., 2018a
	Gold nanorod	RH	0.51 nm/%	0.16%	Zhou et al., 2019
Graphene functionalized microfiber sensors	Graphene-based bottle-shaped cavity	NH_3_	200 kHz/ppm	1ppb	Yao et al., 2017
	Graphene-coated microfiber	Temperature	2.10 dB/°C	0.0005°C	Wang et al., 2018b
	Graphene-oxide-based microfiber-knot resonator	RH	0.0104 nm/%	0.1%	Azzuhri et al., 2018
	Graphene-coated silica microfiber	Magnesium	19.63 dBm/%	0.0038%	Yasin et al., 2018
Optofluidic microfiber sensors	Nanofiber pair	Nanoparticle	/	Single nanoparticle	Yu et al., 2014
	Microfiber-capillary	MicroRNA-let7a	/	1.43 ng/mL	Liang et al., 2017
	Coiled optical nanofiber	Chloramphenicol	/	0.5 ng/L	Mei et al., 2019

#### Future Prospects

In the past years, optical sensing is one of the most active areas in MNF-based optics and technology and has shown great potential for physical, chemical, and biological sensing on micro-/nanoscale. However, the field is still in the initial stage of development, as very few MNF sensors have been moved from laboratory to practical applications. To bridge the gap, a series of issues, including the difficulties in fabrication, functionalization, and package of MNF structures, has to be addressed to significantly enhance the reliability and reproducibility: (1) improving fabrication system to yield MNFs with designed parameters, high precision, and better reproducibility; (2) developing highly repeatable schemes and systems to functionalize MNFs with desired geometries and materials; and (3) packaging MNFs and devices with high robustness and reproducibility. To this end, the rapid development in micro-/nanofabrication technology, together with the emerging robot technology and computer-aided engineering and manufacturing (e.g., 3D printing), may open a route to large-scale fabrication of MNF sensors. Meanwhile, rapid progresses on MNFs with new functional structures and materials, as well as new mechanism or effects for optical sensing, will continue to bestow MNF-based optical sensors with new opportunities including, but not limited to, the following areas.

#### Ultrasensitive Optical Force Sensors at Nanoscale

A noticeable merit of an MNF is its ultralow stiffness and high mechanical flexibility. For example, with the same Young's modulus, the force required to bending a 400-nm-diameter silica MNF into a certain shape is about 100,000 times smaller than a standard silica fiber. Meanwhile, at room temperature, the maximum tolerable strain in a silica MNF is much larger than in standard silica fiber (Brambilla and Payne, 2009, Tong et al., 2003). These mechanical properties are favorable candidate for nanomechanical force transducers for micro-force sensing on micro- or nanoscale (Sirbuly et al., 2015). At the same time, an MNF also has a very low mass or inertia, e.g., a 200-nm-diameter 10-μm-length silica MNF is about 10^−15^ kg in mass or 10 fN in weight, which is comparable with the photon momentum of a 10-μW-power light, making it highly optomechanically sensitive. For example, relying on a nanofiber with white-light interferometry, Yu et al. demonstrated a sensitive and cost-effective scheme to generate, sense, and exploit fN optical force, which paves the way toward mW and fN-optical-force optomechanical devices (Yu et al., 2018a).

#### Novel Biosensing Platform Based on Optofluidic Biolasers

Optofluidic biolasers are emerging as a highly sensitive way to measure changes in biological molecules. Biolasers, which incorporate biological material into the gain medium and contain an optical cavity in a fluidic environment, can use the amplification that occurs during laser generation to quantify tiny changes in biological processes in the gain medium (Fan and Yun, 2014, Gong et al., 2018, Yang et al., 2019). Meanwhile, optical MNF is an ideal platform for lasers (Jiang et al., 2006b) or nanolasers (Xiao et al., 2011b). Also, the surface of a silica MNF can be functionalized with a variety of biomolecules or antibodies. Thus, the fusion of optofluidic laser and MNFs may open a door to novel biosensors with high sensitivity, high selectivity, small footprint, and fast response.

#### Wearable Optical MNF Sensors

**Figure 14:**
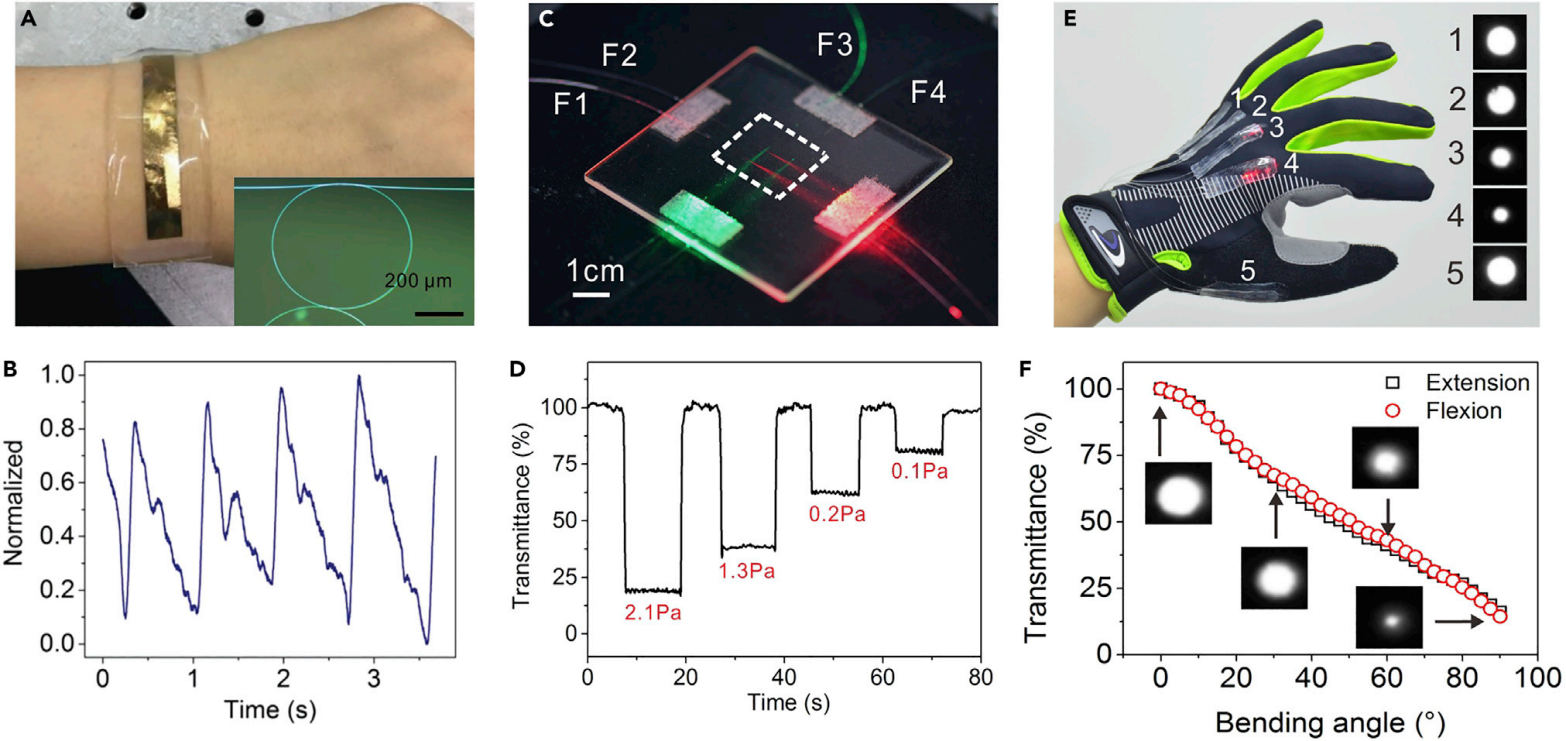
Wearable Optical MNF Sensors (A) Photograph showing a wearable MNF sensor based on an HPMKR. (B) Real-time detection of finger artery pulse. Reprinted with permission from Li et al. (Li et al., 2018b). Copyright 2018 John Wiley & Sons, Inc. (C) Photograph showing an SLWOS consisted by a perpendicularly intersected 2×2 MNF array. (D) Response of a suspended SLWOS to pressure of 2.1, 1.3, 0.2, and 0.1 Pa, respectively. Schematic of testing suspended SLWOS. (E) Photograph showing a five-sensor data glove integrated with five SLWOSs. (F) Bending-angle-dependent output of a typical SLWOS. Reprinted with permission from Zhang et al. (Zhang et al., 2020). Copyright 2019 Institute of Optics and Electronics, Chinese Academy of Sciences.

Electronic skin (E-skin), a class of wearable electronic sensors that mimic the functionalities of human skin, has made remarkable success in applications including health monitoring, human-machine interaction, and electronic-biological interfaces (Hammock et al., 2013). Although E-skin continues to achieve higher sensitivity and faster response, its ultimate performance is fundamentally limited by the nature of low-frequency AC currents. Benefitted from the high mechanical flexibility, together with the tightly confined large fractional evanescent fields, compared with E-skins, an optical-MNF-based wearable sensor can achieve the higher sensitivity with much faster response time and immunity of electromagnetic interference. For example, enabled by a hybrid plasmonic microfiber knot resonator (HPMKR) embedded in a PDMS membrane (Figure 14A), Li et al. (Li et al., 2018b) realized sensing of wrist pulse, respiration, and finger pulse (Figure 14B). Based on an MNF-embedded PDMS patch, Zhang et al. demonstrated a skin-like wearable optical sensor (SLWOS) highly sensitive to micro-deformation. With negligible crosstalk, multiple MNFs can be weaved inside a single SLWOS for spatially resolved 2-dimensional tactile sensing (Figure 14C) with a pressure detection limit down to 0.1 Pa (Figure 14D). Figure 14E shows a five-sensor optical data glove for monitoring the flexion and extension of the metacarpophalangeal (MCP) joints of individual fingers, presenting a monotonic and approximately linear dependence of individual SLWOS output on the bending angle (Figure 14E). We believe the MNF-based wearable sensors may find potentials in human health monitoring, human-machine interfaces, and artificial intelligence.

#### Challenges

In the past decades, we have witnessed the success in optical MNF sensors; however, more challenges may come from both practical applications and theoretical research. Firstly, as the fractional power outside the MNF depends on its diameter and the surface coating, how to routinely draw MNF with high precision and functionalize MNF structures with high repeatability are the keys to scalable fabrication of MNF sensors. Secondly, as an open sensitive structure relying on evanescent fields in the vicinity of the surface, how to protect the sensitive MNF element from environmental contamination is usually required. Although a number of approaches, including integrated with optofluidic devices and embedded in low-index polymers, have been successfully developed for packaging MNF structures in recent years, the challenge remains in many cases since extremely high cleanness is typically required for ultrasensitivity sensors, especially when they are designed for long-term operation. Thirdly, as mentioned before, one outstanding advantage of a low-loss waveguiding MNF is its potential to tightly confine large fractional evanescent fields with size comparable with that of a particle-like sample (e.g., protein and many other bio-molecules). Although single-nanoparticle-level sensitivity has been proved (Wang et al., 2007, Yu et al., 2018b), pushing the detection limit of an MNF sensor to single-molecule level remains challenging.

### Conclusions

So far we have reviewed the fabrication, functionalization, optical properties, and sensing applications of MNFs. Typical MNF-based sensing structures, including biconical MNFs, MNF couplers, Mach-Zehnder interferometers, optical gratings, and circular microcavities, are summarized. Categorized by hybrid sensing structures, advanced sensing applications based on functionalized polymer MNFs, metallic-nanostructure-activated MNFs, graphene-decorated MNFs, and optofluidic MNFs are also reviewed. To date, MNF has been emerging as an interdisciplinary platform for exploring novel sensing technology on micro- or nanoscale. The merging of fiber optics, photonics, chemistry, biology, and material science will continue to open up new opportunities in broad areas ranging from nanophotonics, plasmonics, to optofluidics, which may be readily applied for MNF-based optical sensing. At the same time, we have also discussed future prospects and challenges of this field. We hope that some points in this review can be helpful or realized in future studies.
